# High Serum Phosphorus Level Is Associated with Left Ventricular Diastolic Dysfunction in Peritoneal Dialysis Patients

**DOI:** 10.1371/journal.pone.0163659

**Published:** 2016-09-23

**Authors:** Min Ye, Na Tian, Yanqiu Liu, Wei Li, Hong Lin, Rui Fan, Cuiling Li, Donghong Liu, Fengjuan Yao

**Affiliations:** 1 Department of Ultrasound, The First Affiliated Hospital of Sun Yat-sen University, Guangzhou, PR China; 2 Department of Nephrology, The First Affiliated Hospital of Sun Yat-sen University, Guangzhou, PR China; 3 Department of Nephrology, General Hospital of Ningxia Medical University, Yinchuan, PR China; Universidade Estadual Paulista Julio de Mesquita Filho, BRAZIL

## Abstract

**Objectives:**

We initiated this study to explore the relationships of serum phosphorus level with left ventricular ultrasound features and diastolic function in peritoneal dialysis (PD) patients.

**Methods:**

174 patients with end-stage renal disease (ESRD) receiving PD were enrolled in this retrospective observational study. Conventional echocardiography examination and tissue Doppler imaging (TDI) were performed in each patient. Clinical information and laboratory data were also collected. Analyses of echocardiographic features were performed according to phosphorus quartiles groups. And multivariate regression models were used to determine the association between serum phosphorus and Left ventricular diastolic dysfunction (LVDD).

**Results:**

With the increase of serum phosphorus levels, patients on PD showed an increased tissue Doppler-derived E/e’ ratio of lateral wall (*P* < 0.001), indicating a deterioration of left ventricular diastolic function. Steady growths of left atrium and left ventricular diameters as well as increase of left ventricular muscle mass were also observed across the increasing quartiles of phosphorus, while left ventricular ejection fraction remained normal. In a multivariate analysis, the regression coefficient for E/e’ ratio in the highest phosphorus quartile was almost threefold higher relative to those in the lowest quartile group. And compared with patients in the lowest phosphorus quartile (<1.34 mmol/L) those in the highest phosphorus quartile (>1.95 mmol/L) had a more than fivefold increased odds of E/e’ ratio >15.

**Conclusions:**

Our study showed an early impairment of left ventricular diastolic function in peritoneal dialysis patients. High serum phosphorus level was independently associated with greater risk of LVDD in these patients. Whether serum phosphorus will be a useful target for prevention or improvement of LVDD remains to be proved by further studies.

## Introduction

Cardiovascular disease (CVD) is the leading cause of death among patients with ESRD [1–2], accounting for more than 50% of overall mortality [3]. The cardiovascular mortality risk in patients with ESRD starting dialysis was estimated to be 15-fold higher compared with the age-, race-, and sex-matched general population [4]. Left ventricular diastolic dysfunction occurs frequently in chronic kidney disease (CKD) patients and even in patients with early stages of chronic kidney dysfunction [5]. And it has been proved as predictive of a worse cardiovascular (CV) prognosis in this population [6–8].

Patients with ESRD on peritoneal dialysis manifest a high prevalence of mineral metabolism disorders, including elevated concentrations of serum phosphorus, parathyroid hormone (PTH), and decreased calcium. There were a large number of studies focusing on the relationship between mineral metabolism disturbances and mortality risk in dialysis patients [9–12]. Although most studies were carried out in hemodialysis patients, hyperphosphatemia has been proved as a well-established risk factor for all-cause mortality and cardiovascular death[13–17], even in pre-dialysis patients[18] or general population[19–21]. There was also evidence about the significant association between high serum phosphorus with CV event risks (including congestive heart failure, myocardial infarction, transient ischaemic attack or stroke) [22–23]. In addition, the relationship between serum phosphorus and cardiovascular Events has been further confirmed by studies conducted in patients without CKD or CVD [24–25].

LV diastolic dysfunction detected via an increased early diastolic mitral inflow velocity (E) to early mitral annular diastolic velocity (e’) ratio (E/e’ ratio) has been proved as a marker for increased risk of cardiovascular events and all-cause mortality in CKD patients [26–29]. However, until now, there are no data evaluating the association of serum phosphorus level with LV diastolic dysfunction in peritoneal dialysis patients. Therefore, we initiated this study to explore the relationship of serum phosphorus with left ventricular ultrasound features and function in peritoneal dialysis patients.

## Materials and Methods

### Study population

Patients with ESRD receiving continuous ambulatory peritoneal dialysis for more than 3 months in a single PD center of the First Affiliated Hospital of Sun Yat-sen University between July 2013 and April 2014 were eligible for inclusion in the study. Additional inclusion criteria were: conventional laboratory measurements including serum phosphorus, and echocardiography were performed. Those who had a history of coronary disease or myocardial infarction, cardiomyopathy, significant arrhythmias, severe mitral valve disease, pericardial disease, or congenital heart disease were excluded. Clinical information, including demographic data, prior medical history (such as hypertension, diabetes, and etiology of CKD) and risk factors for CVD (both traditional and uremia-related risk factors) were collected from medical records. Baseline clinical assessment, including height, weight, blood pressure, and pulse were also recorded. Body mass index (BMI) was calculated as weight (kg) divided by height square (m)^2^. The study protocol was approved by the ethical review board of the First Affiliated Hospital of Sun Yat-sen University and all patients gave written informed consent. The study was registered with the number NCT02000128.

### Laboratory Measurements

All blood samples were drawn from the medial cubital vein in the morning after an overnight fasting of at least 8 h. Biochemical parameters, including serum phosphorus, calcium, intact parathyroid hormone (iPTH), serum albumin, cholesterol, triglyceride, creatinine, pro-brain natriuretic peptide (ProBNP) and hemoglobin (Hb) were measured at the clinical biochemistry laboratory of the First Affiliated Hospital of Sun Yat-sen University. Analytic coefficients of variation were <5%.

### Echocardiography

Conventional two-dimensional echocardiography was performed according to guidelines using a commercial ultrasound system (Vivid 7, GE Health Medical, Milwaukee, WI, USA) supported with a multi-frequency transducer (M3S 1.7/3.4 MHz). All echocardiographic measurements were carried out according to American Society of Echocardiography (ASE) criteria [30] by the same independent echocardiologist. Left ventricular mass (LVM) was calculated according to the ASE–recommended cube formula from linear dimensions and indexed to body surface area (LVMI). Left ventricular hypertrophy (LVH) was defined as LVMI >134 g/m^2^ in men and >110g/m^2^ in women [30]. Left ventricular ejection fraction (LVEF) was measured from the parasternal long axis view using Teichholz method [30]. Transmitral inflow was measured in apical four-chamber view using pulsed wave Doppler recordings at the mitral valve leaflet tips. Peak velocities of early filling (E), atrial filling (A), and the E/A ratio were recorded. Early mitral annulus velocity (e’) was measured at the lateral portion of the mitral annulus in an apical four chamber view using a tissue Doppler technique with a Nyquist limit of 15 cm/s. E/e’ ratio was calculated as peak velocities of early filling (E) divided by e’. In our study, LVEF >55% was defined as normal ventricle systolic function. LV filling pressure was considered to be elevated when E/e’ >15 and normal when E/e’ <8. LV diastolic dysfunction was defined when lateral e’ <10 cm/s and E/e’ >15 [31–33].

### Statistical analysis

All continuous and normally distributed variables are presented as mean ± standard deviation (SD), while categorical variables were described as proportions. Shapiro-Wilk test was used to assess the normality of variables’ distribution. Homogeneity of variance was checked using Levene’s test. In case of normal distribution and homogeneity of variance, between-group differences were analyzed by ANOVA with LSD post hoc test, while if at least one of the above mentioned criteria was not met, Kruskal–Wallis with Mann–Whitney U post hoc test were used. Pearson and partial Pearson test were used for correlation analysis between serum phosphorus and echocardiographic parameters. Multivariate linear regression models were used to explore the association between serum phosphorus and E/e’ ratio. And multiple logistics regression models were used to analyze the association between serum phosphorus levels and risk of E/e’ ratio >15. Serum phosphorus was considered as both continuous and categorical variables in the multivariate regression analyses. Potential confounders were retained in the final model if they were prior considered important (age, BMI, blood pressure, and medical history of DM and hypertension) or their inclusion resulted in a >10% change in the estimate. Unadjusted and adjusted regression coefficients or odds ratios (ORs) were reported along with their 95% confidence intervals (CI) and statistical significance was set at P<0.05. All analyses were performed with Statistical Package for Social Sciences (SPSS), Version 13.0 (SPSS Inc., Chicago, IL, USA).

## Results

### Clinical, Biochemical and Echocardiographic Characteristics by Serum Phosphorus category

**Fig 1:**
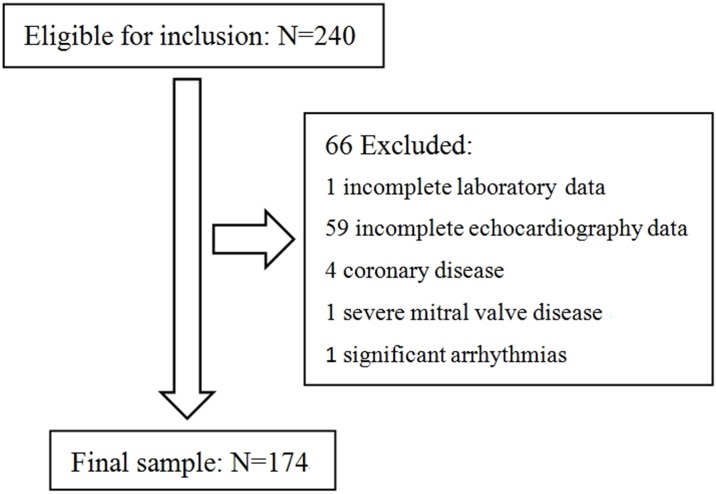
Study flow, including patient enrollment, and exclusions based on patients’ characteristics.

We reviewed 240 patients’ medical records resulting in a final sample of 174 PD patients (Fig 1). Out of the 240 patients deemed to be eligible for our study, 60 had either incomplete laboratory or echocardiography data. An additional six patients had either coronary disease, severe mitral valve disease or significant arrhythmias and were excluded from the analysis. We stratified patients into four groups according to the quartiles of serum phosphorus level. Analyses of baseline characteristics of patients were presented in Table 1. The mean age of the 174 patients was 48.5±15.8 year and 48.9% were male. The mean serum phosphorus concentration was 1.7±0.5 mmol/L. Compared with participants with lower concentrations of serum phosphorus levels, those with higher concentrations were more likely to have higher BMI. There was a trend of higher levels of serum creatinine (*P* for trend <0.001), iPTH (*P* for trend <0.001), and ProBNP (*P* for trend <0.001) across increasing quartiles of phosphorus, although not all the difference between groups reach statistical significance. However, hemoglobin decreased across increasing phosphorus quartiles (*P* < 0.001). All other biochemical characteristics were not significantly different according to phosphorus quartile groups.

**Table 1:** Baseline characteristic of peritoneal dialysis patients by quartiles of serum phosphorus (N = 174).

Characteristic	Serum phosphorus quartiles (mmol/L)				*P*	*P*-trend
	<1.34 (n = 45)	1.34–1.61 (n = 42)	1.62–1.95 (n = 44)	>1.95 (n = 43)		
**Basic charateristics**						
Male	25(55.6)	18(42.9)	21(47.7)	21(48.8)	0.703	0.639
Age (y)	50.4±15.8	47.5±17.8	48.9±15.6	48.5±15.8	0.765	0.432
BMI (kg/m^2^)	20.9±3.0	21.8±2.8	22.2±3.0	23.6±3.1‡§#	0.001	<0.001
Dialysis duration (m)	25.8±24.8	32.8±21.4	31.3±23.7	39.3±21.3	0.056	0.013
Prevalent diabetes	14(31.1)	9(21.4)	9(20.5)	26(39.5)	0.161	0.456
Prevalent hypertension	45(100)	41(97.6)	43(97.7)	41(95.3)	0.554	0.191
Systolic BP (mmHg)	139.9±17.0	138.3±20.2	144.5±19.8	142.3±19.8	0.471	0.307
Diastolic BP (mmHg)	83.1±12.8	84.0±13.2	86.6±12.7	82.2±13.3	0.440	0.994
**Laboratory parameter**						
Creatinine (umol/L)	783.1±266.6	916.1±217.2‡	1057.0±284.0‡§	1252.2±274.8‡§#	<0.001	<0.001
Triglycerides (mmol/L)	1.3(0.9–2.5)	1.1(0.9–1.8)	1.2(0.8–1.7)	1.4(1.0–1.8)	0.388	0.267
TCHO (mmol/L)	5.1±1.0	4.7±1.2	5.1±1.1	5.1±1.3	0.282	0.523
HDL-C (mmol/L)	1.1±0.4	1.1±0.3	1.2±0.3	1.0±0.3	0.121	0.216
LDL-C (mmol/L)	2.9±0.8	2.6±0.8	2.9±0.9	2.9±0.9	0.223	0.390
Calcium† (mmol/L)	2.3±0.2	2.4±0.2	2.4±0.2	2.4±0.2	0.255	0.527
iPTH (pg/mL)	300.6(202.2–459.3)	415.8(272.0–619.1)‡	459.2(362.6–770.5)‡	609.6(482.2–1054.4)‡§#	<0.001	<0.001
Albumin (g/L)	37.1±4.1	38.3±2.9	37.8±3.5	38.6±3.5	0.196	0.094
Hb (g/L)	112.9±19.7	113.8±15.3	109.4±16.4	98.1±20.8‡§#	<0.001	<0.001
hsCRP (mg/L)	1.7(0.5–5.4)	0.9(0.3–2.1)	1.9(0.4–5.1)	2.4(1.1–4.1)	0.338	0.179
ProBNP (pg/mL)	2394.0(1126.4–3623.3)	2020.0(1059.0–8269.5)	6956.0(2275.0–15979.0)	12985.0(2961.0–35000.0)‡§#	<0.001	<0.001

^†^Corrected for serum albumin concentration (correction formula: albumin-corrected calcium(mmol/L) = total calcium(mmol/L) + 0.02 × (40–albumin g/l))[34].

BP = blood pressure; iPTH = intact parathyroid hormone; TCHO = total cholesterol; HDL-C = high-density lipoprotein cholesterol; LDL-C = low-density lipoprotein cholesterol; Hb = hemoglobin; hsCRP = high sensitivity C reactive protein; ProBNP = pro-brain natriuretic peptide.

^‡^ *P* < 0.05 compared with quartile 1 (<1.34);

^§^ *P* < 0.05 compared with quartile 2 (1.34–1.61);

^#^ *P* < 0.05 compared with quartile 3 (1.62–1.95).

Comparisons of echocardiographic characteristics among the four groups were detailed in Table 2. Steady growths of left atrium (LA) diameter, diameters of left ventricular (LV) end diastole and systole as well as increase of LV wall thickness were observed across the increasing quartiles of serum phosphorus in PD patients. In addition, left ventricular muscle mass (LVM) and LVMI were significantly increased in the highest quartile of phosphorus with respect to the lower quartiles (*P* < 0.001). Furthermore, LV diastolic function were significantly deteriorated in the highest quartile of phosphorus compared to the lowest quartile (E, 67.8±22.5 vs 95.3±31.3, *P* < 0.001; E/A ratio, 0.84±0.32 vs 1.11±0.66, *P* = 0.018; E/ e’ ratio, 9.2±3.7 vs 13.4±6.8, *P* < 0.001), while indicator of LV systolic function (ejection fraction) was almost within normal range and quite similar among the four groups (*P* = 0.180).

**Table 2:** Echocardiographic and Doppler parameters of peritoneal dialysis patients by quartiles of serum phosphorus (N = 174).

Characteristic	Serum phosphorus quartiles (mmol/L)				*P*	*P*-trend
	<1.34 (n = 45)	1.34–1.61 (n = 42)	1.62–1.95 (n = 44)	>1.95 (n = 43)		
LA (mm)	36.8±6.3	39.1±6.5	39.7±5.1‡	43.2±7.5‡§#	<0.001	<0.001
LVDs (mm)	11.8±1.9	11.7±2.2	12.6±2.2	13.2±2.6‡§#	0.006	0.002
LVDd (mm)	50.0±5.8	51.5±7.6	52.2±7.3	55.4±8.0‡§#	0.006	0.001
IVSd (mm)	31.7±5.4	32.6±6.8	32.8±5.6	36.7±9.5‡§	0.004	0.001
LVPWd (mm)	10.6±1.8	10.4±1.8	11.3±1.8§	12.1±2.5‡§	<0.001	<0.001
LVEF (%)	65.0±7.3	64.7±9.3	66.1±6.8	61.8±11.6	0.142	0.180
LVM (g)	218.6±68.9	226.3±84.8	254.0±78.6	305.6±109.7‡§#	<0.001	<0.001
LVMI (g/m^2^)	140.1±41.8	144.9±50.4	156.9±41.5	182.0±58.3‡§#	<0.001	<0.001
E (cm/sec)	67.8±22.5	79.7±30.3‡	82.6±26.4‡	95.3±31.3‡§#	<0.001	<0.001
A (cm/sec)	84.1±18.7	86.0±19.3	94.9±18.4‡	93.4±29.7	0.054	0.015
E/A ratio	0.84±0.32	0.97±0.42	0.89±0.29	1.11±0.66‡#	0.032	0.018
e’ (cm/sec)	8.1±3.2	7.9±2.2	7.5±2.2	7.9±2.3	0.761	0.537
E/e’ ratio	9.2±3.7	10.7±4.9	11.5±4.1‡	13.4±6.8‡§	0.002	<0.001

LA = left atrium; LVDs = end-systolic left ventricular diameter; LVDd = end-diastolic left ventricular diameter; IVSd = end-diastolic interventricular septum thickness; LVPWd = end-diastolic left ventricular posterior wall thickness; LVEF = left ventricular ejection fraction; LVM = left ventricular mass; LVMI = Left ventricular mass index; E = early diastolic mitral inflow velocity; A = mitral peak velocity of late filling; e’ = early diastolic lateral mitral annular velocity.

^‡^ *P* < 0.05 compared with quartile 1 (<1.34);

^§^ *P* < 0.05 compared with quartile 2 (1.34–1.61);

^#^ *P* < 0.05 compared with quartile 3 (1.62–1.95).

### The Correlation analysis between serum phosphorus and Echocardiographic parameters

In the correlation analysis, serum phosphorus showed a positive association with echocardiographic parameters studied. High serum phosphorus level was significantly correlated with the increase in both left heart dimensions (LA: *r* = 0.38, *P* < 0.001; LVDd: *r* = 0.30, *P* < 0.001), wall thicknesses (IVSd: *r* = 0.34, *P* < 0.001; LVPWd: *r* = 0.34, *P* < 0.001), and worsening of LV diastolic function (E/ e’ ratio: *r* = 0.37, *P* < 0.001) (Table 3). Furthermore, positive correlations were also found between serum phosphorus and both of LV mass (*r* = 0.44, *P* < 0.001) and LVMI (*r* = 0.38, *P* < 0.001). However, after controlling for traditional and uremia-related CV risk factors, the positive correlations between serum phosphorus and mentioned echocardiographic parameters disappeared, except for E/e’ ratio. After the adjustment, the effect of serum phosphorus on E/e’ ratio modestly weakened but still remained statistically significant (*r* = 0.26, *P* = 0.002).

**Table 3:** Correlation analysis of serum phosphorus and echocardiographic measurements in peritoneal dialysis patients (N = 174).

	Unadjusted correlation coefficient	P value	Adjusted correlation coefficient*	*P* value
E/e’ ratio	0.37	<0.001	0.26	0.002
LA	0.38	<0.001	0.07	0.419
LVDd	0.30	<0.001	-0.02	0.830
IVSd	0.34	<0.001	0.13	0.129
LVPWd	0.34	<0.001	0.08	0.311
LVEF	-0.19	0.011	-0.11	0.181
LVM	0.44	<0.001	0.12	0.162
LVMI	0.38	<0.001	0.10	0.242

*Adjusted for: age, gender, dialysis duration, body mass index, blood pressure, prevalent diabetes, prevalent hypertension, albumin-corrected calcium (correction formula: albumin-corrected calcium(mmol/L) = total calcium(mmol/L) + 0.02 × (40–albumin g/l)), iPTH, serum albumin, TCHO, total triglyceride, HDL-C, LDL-C, hsCRP, serum creatinine and Hb.

### Multivariate Linear Regression analysis between serum phosphorus and E/e’ ratio

In order to further explore the relationship between serum phosphorus level and left ventricular diastolic function, we performed multivariate linear regression analyses of serum phosphorus concentrations with E/e’ ratio. As shown in Table 4, patients with higher phosphorus experienced significantly higher E/e’ ratio (β: 4.06, 95% CI: 2.53, 5.59). The association was attenuated slightly but remained statistically significant, with the adjustments for demographic data and medical history (β for model 1: 3.23, 95% CI: 1.76, 4.70) and other CV risk factors (β for model 2: 2.84, 95% CI: 1.30, 4.38). When taken serum phosphorus as category variables with quartiles for further analysis, the association remained only significant at the highest quartile group. The regression coefficient for E/e’ ratio in the highest quartile group was 3.42 (95% CI: 1.45, 5.39) times higher in model 1 and 2.83 (95% CI: 0.77, 4.89) times higher in model 2, compared with the lowest quartile group. Taken together, these results indicated that elevated serum phosphorus levels may be an independent risk factor for LV diastolic dysfunction in patients receiving peritoneal dialysis.

**Table 4:** Multiple Linear Regression Analysis for the association between serum phosphorus and E/e’ ratio among peritoneal dialysis patients (N = 174).

Serum Phosphorus (mmol/L)	Regression Coefficient		
	Unadjusted	Model 1 [95%CI]*	Model 2 [95%CI]†
<1.34	reference	reference	reference
1.34–1.61	1.51[-0.61,3.63]	1.73[-0.24,3.69]	1.74[-0.29,3.76]
1.62–1.95	2.30[0.20,4.39]	2.21[0.27,4.16]	2.01[0.02,3.99]
>1.95	4.16[2.05,6.27]	3.42[1.45,5.39]	2.83[0.77,4.89]
*P* for trend	<0.001	0.001	0.008
Continuous	4.06[2.53,5.59]	3.23[1.76,4.70]	2.84[1.30,4.38]
*P* value	<0.001	<0.001	<0.001

*Adjusted for: age, gender, dialysis duration, body mass index, systolic blood pressure, diastolic blood pressure, prevalent diabetes, and prevalent hypertension.

^†^Adjusted for model 1 covariates plus albumin-corrected calcium, iPTH, serum albumin, TCHO, total triglyceride, HDL-C, LDL-C, hsCRP, serum creatinine and Hb.

CI = confidence interval.

### Multivariate Logistics Regression analysis between serum phosphorus and risk of E/e’ ratio >15

**Fig 2:**
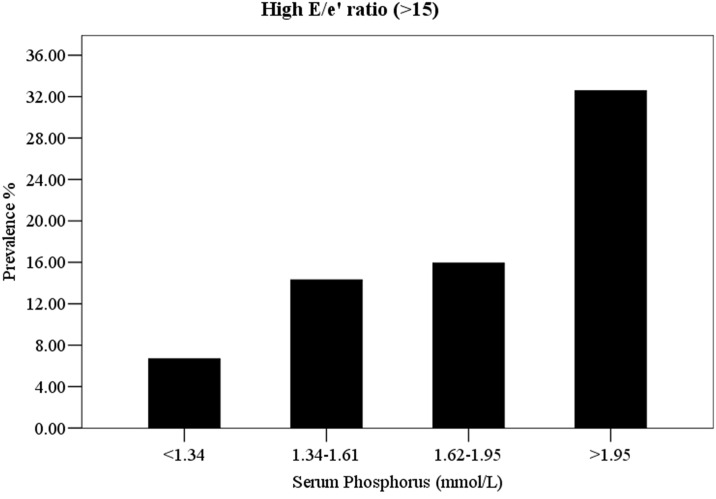
Prevalence of high E/e’ ratio (>15) by quartiles of serum phosphorus among peritoneal dialysis patients (N = 174).

In present study, we further analyze the relationship between serum phosphorus and left ventricular diastolic dysfunction, defined as E/e’ ratio >15. As shown in Fig 2, patients with phosphorus concentrations between 1.34 and 1.95 mmol/L had approximately twice the prevalence of E/e’ ratio >15, while patients with phosphorus concentrations >1.95 mmol/L had more than fourfold the prevalence, compared to patients with phosphorus concentrations <1.34 mmol/L. Table 5 showed the multiple logistic regression analyses of the relationship between serum phosphorus with E/e’ ratio >15. In unadjusted models, the highest risk for E/e’ ratio >15 was observed among patients who had serum phosphorus concentrations >1.95 mmol/L (OR: 6.76, 95% CI: 1.78, 25.65). After demographic and medical history adjustments, those in the highest quartile group were 5.60 (95% CI: 1.44, 21.80) times more likely to develop E/e’ ratio >15, relative to those in the lowest quartile of phosphorus. This association was attenuated only slightly with further adjustment for other CV risk factors (OR: 5.24; 95% CI: 1.34, 20.45). When taken serum phosphorus as continuous variable, each 1 mmol/L increase in phosphorus concentrations was associated with approximately fivefold increased risk for E/e’ ratio >15 in the unadjusted model (OR: 4.97; 95% CI: 2.11, 11.69). And the positive associations remained stable even after adjustments for demographic, medical history and other CV risk factors (OR for model 1: 4.56; 95% CI: 1.88, 11.05; OR for model 2: 4.71, 95% CI: 1.90, 11.63).

**Table 5:** Odds ratios for the association between quartile of serum phosphorus and E/e’ ratio >15 among peritoneal dialysis patients (N = 174).

Serum Phosphorus (mmol/L)	Odds Ratio		
	unadjusted[95%CI]	Model 1 [95%CI]*	Model 2 [95%CI]†
<1.34	reference	reference	reference
1.34–1.61	2.33[0.54,10.00]	2.14[0.49,9.38]	1.69[0.37,7.78]
1.62–1.95	2.65[0.64,10.99]	2.47[0.58,10.54]	2.31[0.54,9.86]
>1.95	6.76[1.78,25.65]	5.60[1.44,21.80]	5.24[1.34,20.45]
*P* for trend	0.003	0.008	0.008
Continuous	4.97[2.11,11.69]	4.56[1.88,11.05]	4.71[1.90,11.63]
*P* value	<0.001	0.001	0.001

*Adjusted for: age, gender, dialysis duration, body mass index, systolic blood pressure, diastolic blood pressure, prevalent diabetes, and prevalent hypertension.

^†^Adjusted for model 1 covariates plus albumin-corrected calcium, iPTH, serum albumin, TCHO, total triglyceride, HDL-C, LDL-C, hsCRP, serum creatinine and Hb.

CI = confidence interval.

## Discussion

In the present study, we analyzed the relationship of serum phosphorus with indices of LV diastolic function in PD patients. And results showed that high phosphorus was independently associated with the impairment of LV diastolic function, characterized by a progressive rise of E/e’ ratio. Ommen S.R et al. proposed that the E/e’ ratio be used as the initial measurement for estimation of LV filling pressures that combines the influence of transmitral driving pressure and myocardial relaxation, particularly in those patients with preserved systolic function. Patients with E/e’ >15 can be classified as having elevated filling pressure [32].

Numerous epidemiological studies conducted over the past two decades have demonstrated that hyperphosphatemia was independently associated with increased cardiovascular event rate and CV mortality in patients on dialysis [15,35–37]. However, the serial cardiac changes in dialysis patients with hyperphosphatemia remained unclear. In present study, both the conventional mitral inflow Doppler and the TDI examinations showed an early deterioration of the LV diastolic function in PD patients with the increase of serum phosphorus. In particular, the LV diastolic function worsened with the increase of serum phosphorous concentrations. We found a trend of higher E/e’ ratio (*P* for trend = 0.008) and increased risk for E/e’ ratio >15 (*P* for trend = 0.008) across increasing quartiles of phosphorus in PD patients. In further regression analyses, our results showed that highest phosphorus quartile (>1.95 mmol/L) was significantly associated with increased risk of E/e’ ratio >15, irrespective of unadjusted and adjusted models. These findings confirmed a positive association between high serum phosphorus levels and LV diastolic dysfunction, in accordance with those obtained by Galetta et al.[38] in uremic patients on hemodialysis.

Data from Kimura et al. [39] suggested that LV diastolic dysfunction in uremic patients may be attributable to changes of left ventricular mass index. While in present study, it's noteworthy that the positive correlations between serum phosphorus and LVMI disappeared after adjustments, while correlations with E/e’ ratio remained significant. These findings indicate that, though LVH was known as an important risk factor for LVDD, the effects of higher phosphorus levels on worsening LV diastolic function were at least partially independent of LVH. The mechanisms underlying the linkage of serum phosphorus and E/e’ ratio may be multifactors, including endothelial dysfunction, accelerated atherosclerosis, increased arterial stiffness and vascular calcification. Excess phosphorus can promote metastatic calcification of coronary artery and valves, as well as other large vascular of the body [40–47]. This calcification that occurred in the cardiovascular system can decrease the LV relaxation and compliance, which directly lead to LV diastolic dysfunction [48–51]. Moreover, the calcification has been proved to positively correlate with generalized arterial stiffening, arteriolosclerosis, higher blood pressure, and decreased coronary perfusion [52–54], all of which in turn may contribute to LV diastolic dysfunction. There were also evidence showing that phosphorus levels were positively associated with subclinical atherosclerosis [55–56] and endothelial dysfunction even in general population [57]. Experimental studies also supported the assumption that phosphorus disorders could acutely increase endothelial dysfunction by impairing vasodilation, leading to large vessel and left ventricular dysfunction [58]. Additionally, animal studies suggested that hyperphosphatemia may increase cardiac fibrosis and aggravate microvascular disease [59], which may also have an adverse impact on LV diastolic function.

To our knowledge, there are no data exploring the relationships between serum phosphorus and echocardiographic features and functions in patients with ESRD receiving peritoneal dialysis. Our study was the first to particularly examine the left ventricular diastolic dysfunction risk associated with serum phosphorus levels in peritoneal dialysis patients. Our results showed that patients with phosphorus concentrations >1.95 mmol/L had more than fourfold the prevalence of E/e’ ratio >15, compared to patients with phosphorus concentrations <1.34 mmol/L. And notably, compared with patients in the lowest phosphorus quartile (<1.34 mmol/L) those in the highest phosphorus quartile (>1.95 mmol/L) had a more than fivefold increased odds of E/e’ ratio >15 (OR: 5.24 [95%CI 1.34, 20.45]).

However, there are several limitations to the present study. First, we were unable to obtain information on residual kidney function, as well as the use of medications that affect phosphorus metabolism, which have been proved as important determinants of CV mortality in patients on dialysis. Moreover, as higher phosphate levels may also be the result of slower peritoneal transporter status [60], the lack of data on adequacy and peritoneal transporter status may also have impact on analyzing the real relationship of serum phosphorus levels and LVDD. Second, we did not assess the associations between serum phosphorus and severity of LVDD via echocardiography, which may have further correlation with CV prognosis. Finally, we selected Chinese patients undergoing PD with strict exclusion criteria for present study. Thus, our results could not be applied to those patients who have myocardial infarction, cardiomyopathy, arrhythmias, mitral valve disease, pericardial disease, or congenital heart disease, which limited its generalizability.

## Conclusions

In conclusion, present study showed that patients with ESRD on peritoneal dialysis have a deterioration of LV diastolic function preceding systolic dysfunction. In particular, we found that higher phosphorus was independently associated with the impairment of LV diastolic function, characterized by a progressive rise in E/e’ ratio. Furthermore, our results showed that patients in the highest quartile category of serum phosphorus have 5.24 times higher risk to develop E/e’ ratio >15, compared to those in the lowest quartile. All these findings are coincident with the important role of phosphorous disorders on cardiovascular damage. However, it remains unclear whether efforts to reduce serum phosphorus can prevent or improve LV diastolic dysfunction in these patients. Hence, future prospective research should be conducted to determine whether the reduction of serum phosphorus can translate into a lower risk of LV diastolic dysfunction.
